# Hearing Sensation Levels of Emitted Biosonar Clicks in an Echolocating Atlantic Bottlenose Dolphin

**DOI:** 10.1371/journal.pone.0029793

**Published:** 2012-01-06

**Authors:** Songhai Li, Paul E. Nachtigall, Marlee Breese, Alexander Ya. Supin

**Affiliations:** 1 Marine Mammal Research Program, Hawaii Institute of Marine Biology, University of Hawaii, Kailua, Hawaii, United States of America; 2 Institute of Ecology and Evolution, Russian Academy of Sciences, Moscow, Russia; 3 Key Laboratory of Aquatic Biodiversity and Conservation of the Chinese Academy of Sciences, Institute of Hydrobiology, Chinese Academy of Sciences, Wuhan, China; Institute of Marine Research, Norway

## Abstract

Emitted biosonar clicks and auditory evoked potential (AEP) responses triggered by the clicks were synchronously recorded during echolocation in an Atlantic bottlenose dolphin (*Tursiops truncatus*) trained to wear suction-cup EEG electrodes and to detect targets by echolocation. Three targets with target strengths of −34, −28, and −22 dB were used at distances of 2 to 6.5 m for each target. The AEP responses were sorted according to the corresponding emitted click source levels in 5-dB bins and averaged within each bin to extract biosonar click-related AEPs from noise. The AEP amplitudes were measured peak-to-peak and plotted as a function of click source levels for each target type, distance, and target-present or target-absent condition. Hearing sensation levels of the biosonar clicks were evaluated by comparing the functions of the biosonar click-related AEP amplitude-versus-click source level to a function of external (in free field) click-related AEP amplitude-versus-click sound pressure level. The results indicated that the dolphin's hearing sensation levels to her own biosonar clicks were equal to that of external clicks with sound pressure levels 16 to 36 dB lower than the biosonar click source levels, varying with target type, distance, and condition. These data may be assumed to indicate that the bottlenose dolphin possesses effective protection mechanisms to isolate the self-produced intense biosonar beam from the animal's ears during echolocation.

## Introduction

Since echolocation studies on dolphins were initiated in the early 1950's [1], [2], all of the odontocetes (toothed whales, including dolphins and porpoises) so far investigated have been demonstrated to possess sophisticated echolocation systems and produce highly directional biosonar clicks [3]–[6] used for navigation, environmental investigation, and foraging [7]–[13]. The bottlenose dolphin (*Tursiops truncatus*) was known to be able to produce biosonar clicks with peak-to-peak source levels (i.e., sound pressure levels at 1 m in front of the sound generation structures) up to 228 dB re: 1 μPa [4]. Presumably, the intense emitted clicks with peak-to-peak sound pressure levels over 220 dB re: 1 μPa referenced to a measurement 1 m in front of the animal's head might cause forward masking of the comparatively weak echoes [14] during echolocation. However, the high sound pressure levels of the emitted clicks in front of the animal's head do not necessarily mean that the sound levels perceived by the animal are similarly intense both because of the high directionality of the emitted clicks [3]–[6] and likely neurological suppression of the hearing of the outgoing signals as discovered in echolocating bats [15], [16]. Despite the fact that the biosonar of odontocetes has been investigated for over half a century, little is known about how, and how much, the animals respond to their own emitted clicks during echolocation, except the knowledge learned from a single false killer whale *Pseudorca crassidens* [17].

By recording the brain auditory evoked potentials (AEPs) during echolocation of a false killer whale and comparing the emitted click-related AEP amplitudes to the AEP amplitudes evoked by external “whale-like” clicks in a free acoustic field, Supin and his colleagues demonstrated that the AEP sensation levels of the whale's hearing of its own emitted clicks was approximately −20 to −37 dB relative to the source levels of the biosonar clicks [18]. Both acoustic shadowing mechanisms based on head anatomy and functional regulation of hearing sensitivity may have been responsible for the low sensation levels of self-heard emitted clicks [18]. However, the research was conducted on a single animal of one species, and it is unknown how widely the data and the explanation may be expanded to other species.

In the present study, we investigated the AEP sensation levels of self-heard emitted clicks during echolocation in an Atlantic bottlenose dolphin using the same AEP protocol and in the same experimental facility as those in the study by Supin et al. [18]. The difference of the dolphin biosonar behavior between target-present and target-absent conditions was also examined and discussed.

## Results

### Biosonar clicks

**Figure 1:**
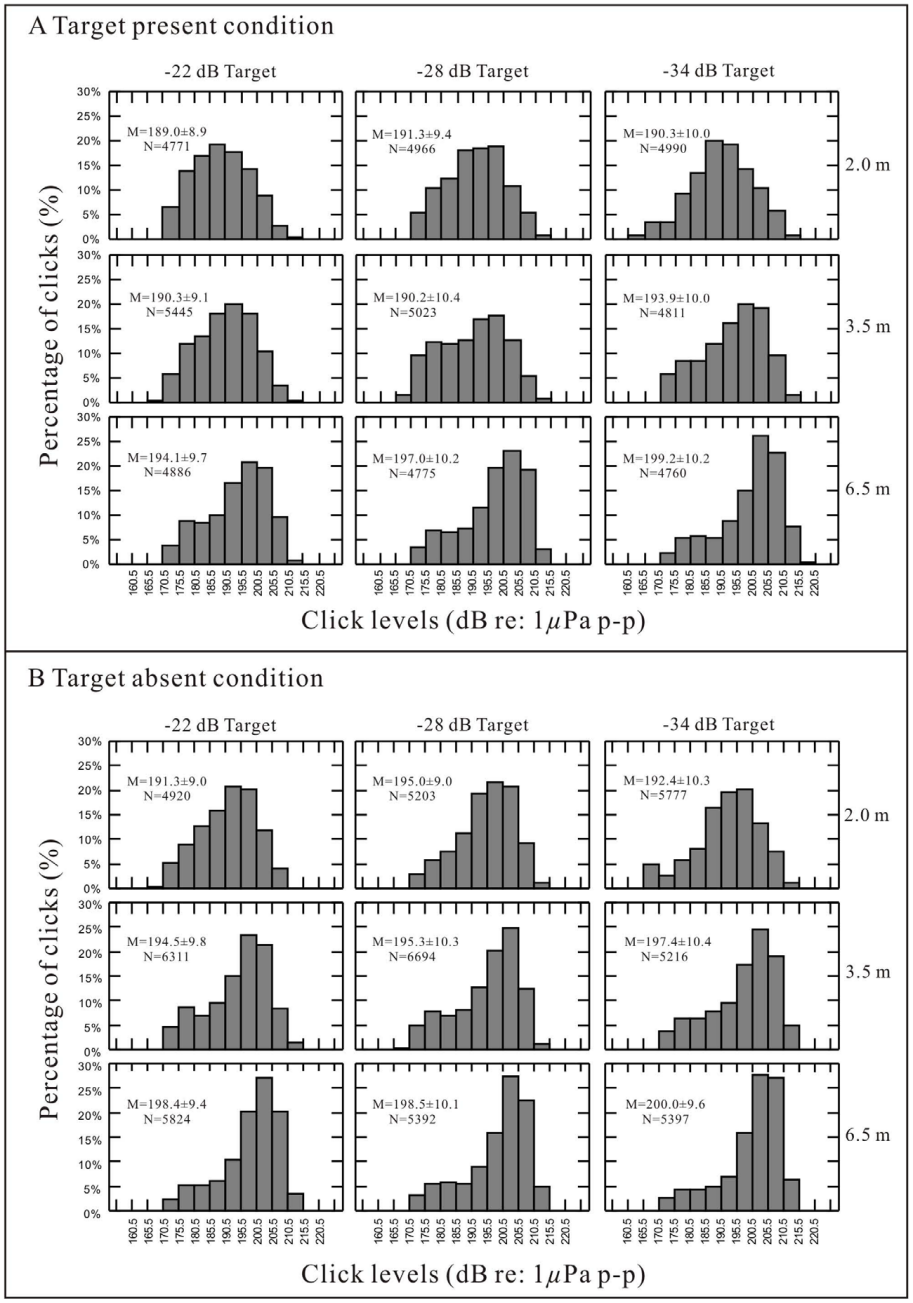
Distributions of emitted click SLs for different target types, distances, and target-present (a) and target-absent (b) conditions. M is the mean value ± s.d. of the click levels, N is number of collected clicks.

Sound pressure levels of the dolphin's biosonar clicks measured at approximately 1.45 m from the animal's nasal sacs were within a range of 160 to 215 dB re 1 μPa peak-to-peak. Assuming that the source levels (SLs), i.e. the sound pressure levels at 1 m from the animal's nasal sacs, are approximately (with 0.5-dB tolerance) 3 dB higher, the SL range was estimated to be 164 to 217 dB re 1 μPa peak-to-peak (Table 1 and Fig. 1). The distributions of the click SLs are presented in Fig. 1 for different target strengths, target distances, and both target-present (Fig. 1a) and target-absent (Fig. 1b) conditions. Mean values (± standard deviations, SD) of the click SLs and number of clicks analyzed are indicated in each panel. The histogram apices of the emitted click SLs gradually improved as the target distances changed from 2 to 6.5 m and target strengths changed from −22 to −34 dB in both the target-present (Fig. 1a) and target-absent (Fig. 1b) conditions, i.e., the animal was inclined to produce louder biosonar clicks to detect further or smaller targets. A comparison of target conditions showed that for all the examined target types and distances, the mean values of the biosonar click SLs in the target-absent conditions were significantly higher than those in the target-present conditions (Fig. 1; T-test for independent samples, *p*<0.05, Table 1), indicating that the animal tended to produce louder clicks in the target-absent conditions.

**Table 1:** Basic statistics of the recorded click source levels (SLs) and comparison between target-present and target-absent conditions (T-test for independent samples, i.e., variables were treated as independent samples).

Target	Target distance (m)	Target present condition	Click SLs (dB re: 1 μPa peak-to-peak)				P-value (T-test)
			Range	Mean	S.D.	Number of clicks	
−22 dB Target	2.0	present	170.8–213.7	**189.0**	8.9	4771	p<0.0005
		absent	169.8–211.6	**191.3**	9.0	4920	
	3.5	present	169.9–214.3	**190.3**	9.1	5445	p<0.0005
		absent	170.9–217.9	**194.5**	9.8	6311	
	6.5	present	171.8–212.7	**194.1**	9.7	4886	p<0.0005
		absent	171.7–214.8	**198.4**	9.4	5824	
−28 dB Target	2.0	present	170.7–214.5	**191.3**	9.4	4966	p<0.0005
		absent	171.0–214.9	**195.0**	9.0	5203	
	3.5	present	169.1–215.3	**190.2**	10.4	5023	p<0.0005
		absent	169.9–215.1	**195.3**	10.3	6694	
	6.5	present	170.6–214.9	**197.0**	10.2	4775	p<0.0005
		absent	170.5–215.4	**198.5**	10.1	5392	
−34 dB Target	2.0	present	164.0–212.0	**190.3**	10.0	4990	p<0.0005
		absent	165.4–215.7	**192.4**	10.3	5777	
	3.5	present	170.8–215.3	**193.9**	10.0	4811	p<0.0005
		absent	170.8–216.8	**197.4**	10.4	5216	
	6.5	present	171.3–216.7	**199.2**	10.2	4760	p<0.0005
		absent	171.0–215.5	**200.0**	9.6	5397	

**Figure 2:**
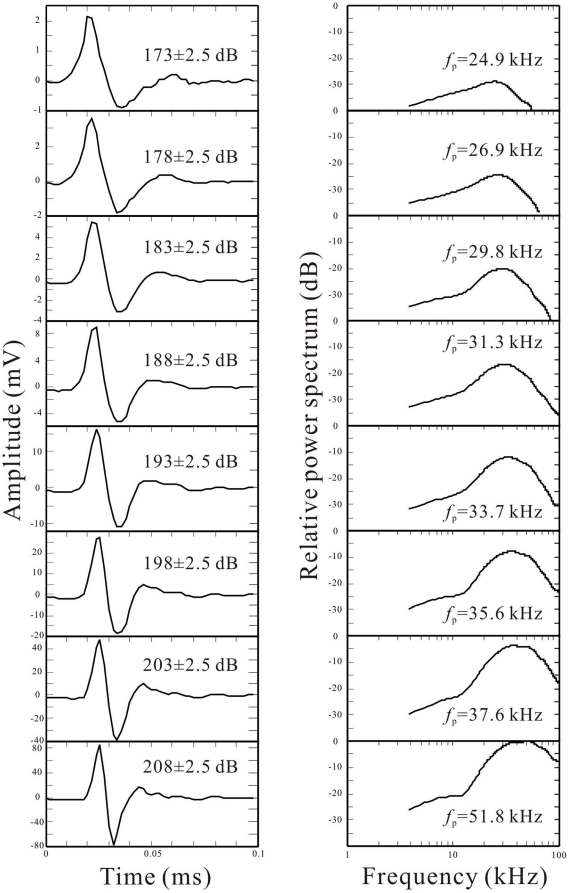
Averaged waveforms and spectra of the transmitting biosonar clicks. (a) Waveforms of the clicks were sorted by peak-to-peak amplitude in 5-dB bins and averaged within each bin. Peak-to-peak click source levels are indicated near the waveforms. (b) Power spectra of the corresponding waveforms. Peak frequencies (*f*_p_) are shown with the spectra.

Examples of averaged click waveforms and spectra are presented in Fig. 2 after averaging the clicks in each 5-dB bin of the click SLs, from 173±2.5 to 208±2.5 dB. Fig. 2 shows that the peak frequencies of the averaged clicks ranged from around 25 kHz at the lowest click SLs to over 50 kHz at the highest SLs.

### Biosonar click-related AEP

**Figure 3:**
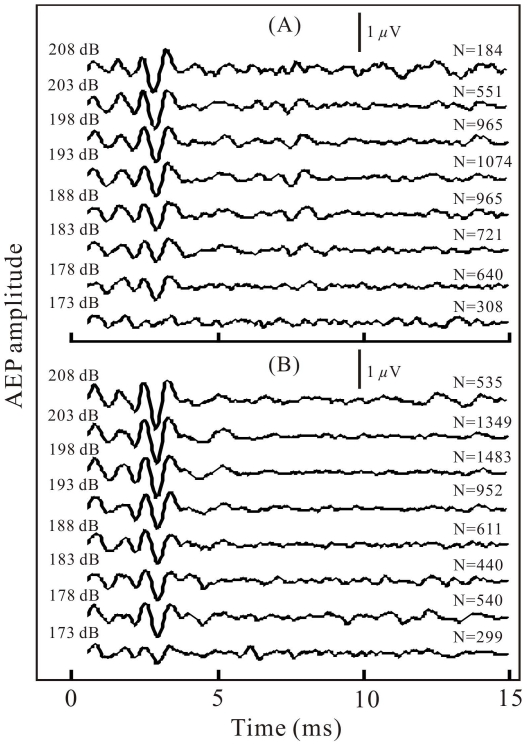
Examples of AEP responses to emitted biosonar clicks. The AEPs were presented after averaging AEP records within each 5-dB bin of the corresponding click SLs in target-present (a) and target-absent (b) conditions. The 5-dB bins (±2.5 dB) of click SLs are indicated near the records in dB *re* 1 μPa. N—number of averaged records. Note that nearly all of the averaged AEP responses had an AEP waveform locating between 1.5 and 3.5 ms in the records well exceeding noise level, consisting of a couple of alternative positive-negative short waves (each shorter than 1 ms). The zero point of the time scale corresponds to the time point when the hydrophone (h in Fig. 9) picked up the clicks and triggered the AEP recordings.

The biosonar click-related AEP waveforms corresponding to certain level bins of the emitted biosonar clicks were extracted after the sorting, averaging, and filtering procedures described in the materials and methods, for each examined target and at each tested distance. Examples of the averaged and filtered AEP recordings are shown in Fig. 3 for both target-present (Fig. 3a) and target-absent (Fig. 3b) conditions, which were extracted from the echolocation tasks during the detection of the −22 dB target at 3.5-m distance. For each averaged AEP record, the corresponding biosonar-click source level (with a tolerance of ±2.5 dB) was assigned at the left side, and the number of AEP records used for averaging was labeled at the end. As shown in Fig. 3, nearly all of the records had an emitted click-related AEP waveform well exceeding the noise level, even in the case where the number of averaged AEP records was less than 200. The click-related AEP waveform was characterized by a couple of alternative positive-negative short waves (each shorter than 1 ms) located between 1.5 and 3.5 ms after the triggering. In both the target-present (Fig. 3a) and target-absent (Fig. 3b) conditions, the amplitudes of the click-related AEP waveforms appeared to increase along with the biosonar-click source levels. The relatively smaller AEP response waveforms located approximately between 7 and 9 ms after the triggering for the recordings in the target-present conditions were the animal's AEP responses to the target echoes at 3.5-m target distance (Fig. 3a).

### AEPs evoked by external “dolphin-like” clicks

**Figure 4:**
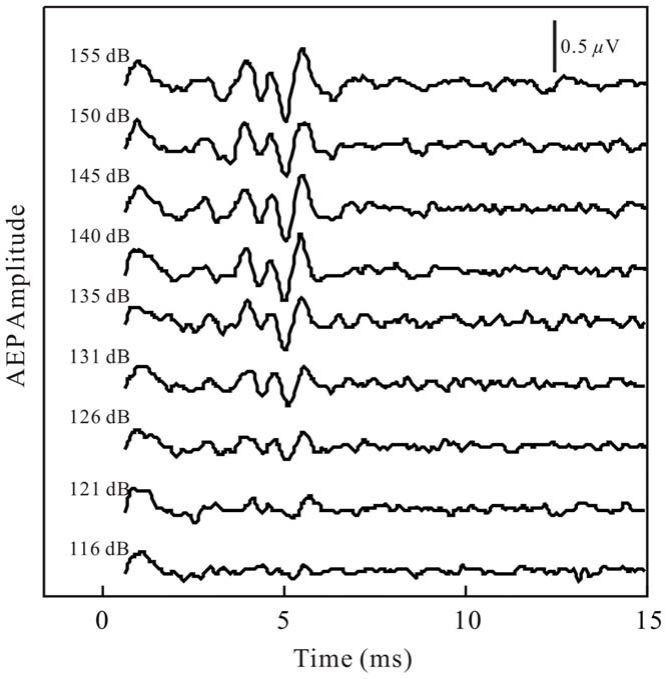
AEP records to external “dolphin-like” clicks. The click peak-to-peak SPLs are indicated near the records in dB *re* 1 μPa. Note that the AEP waveforms are similar to the AEP waveforms in Fig. 3.

**Figure 5:**
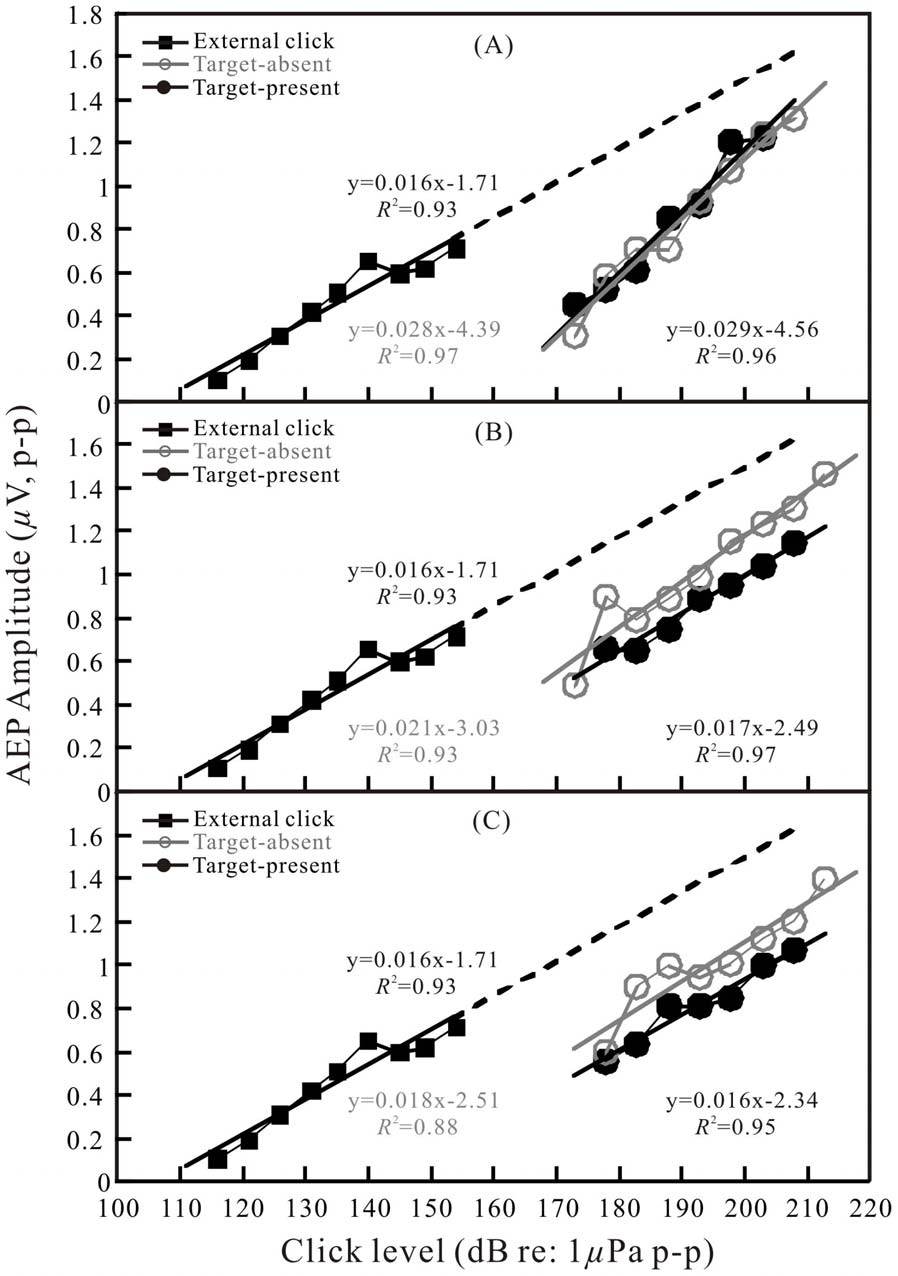
AEP amplitude-versus-click level functions. Comparison between functions of the biosonar click-related AEP amplitude-versus-click SL and the external click-related AEP amplitude-versus-click SPL for the target with −22-dB target strength at distances of 2 (a), 3.5 (b), and 6.5 m (c), respectively, under both target-present and target-absent conditions. For each function, a least-square linear regression line, the corresponding equation and the correlation coefficient are indicated.

**Figure 6:**
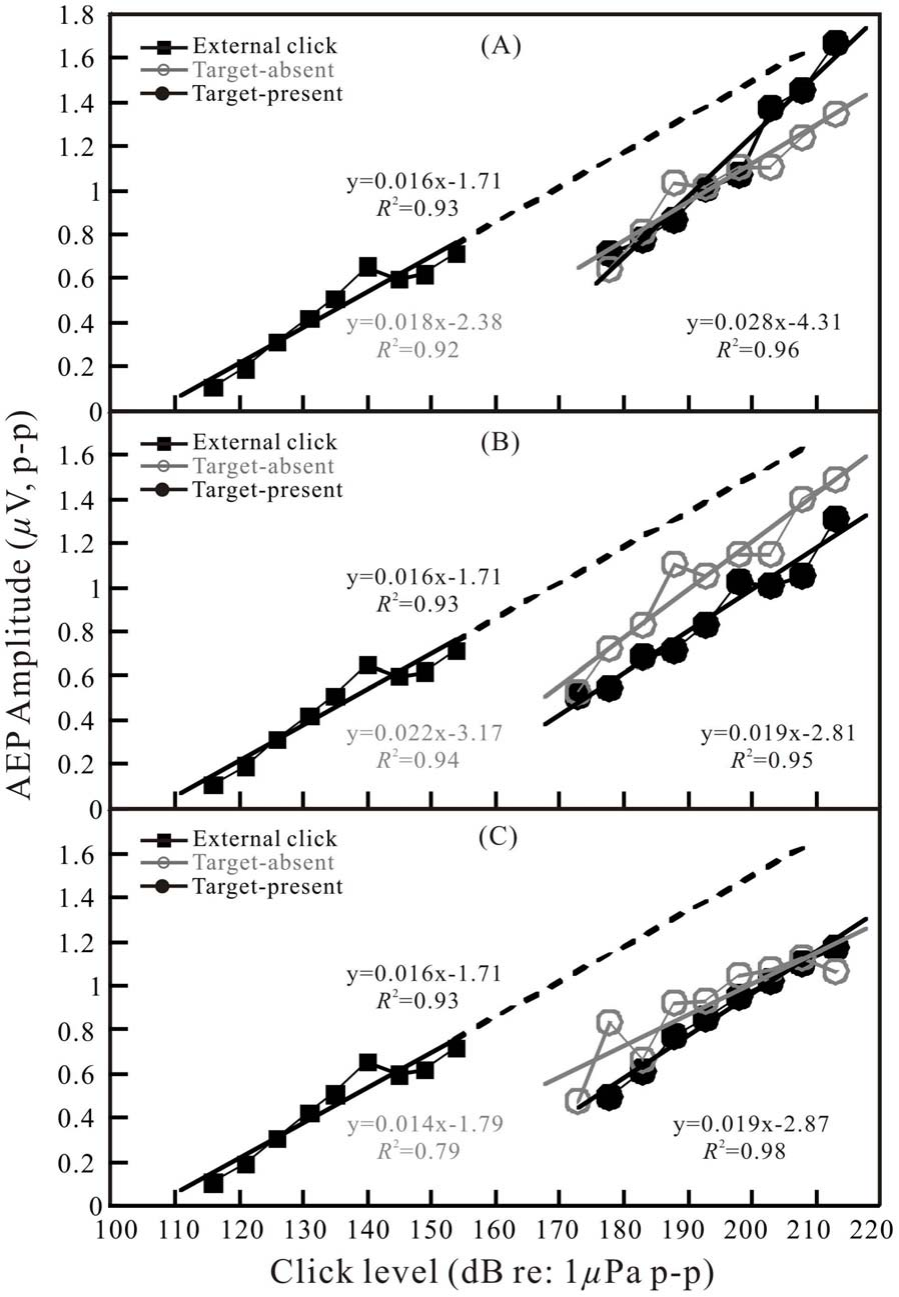
Same as in **Fig. 5** but for the target with −28-dB target strength.

**Figure 7:**
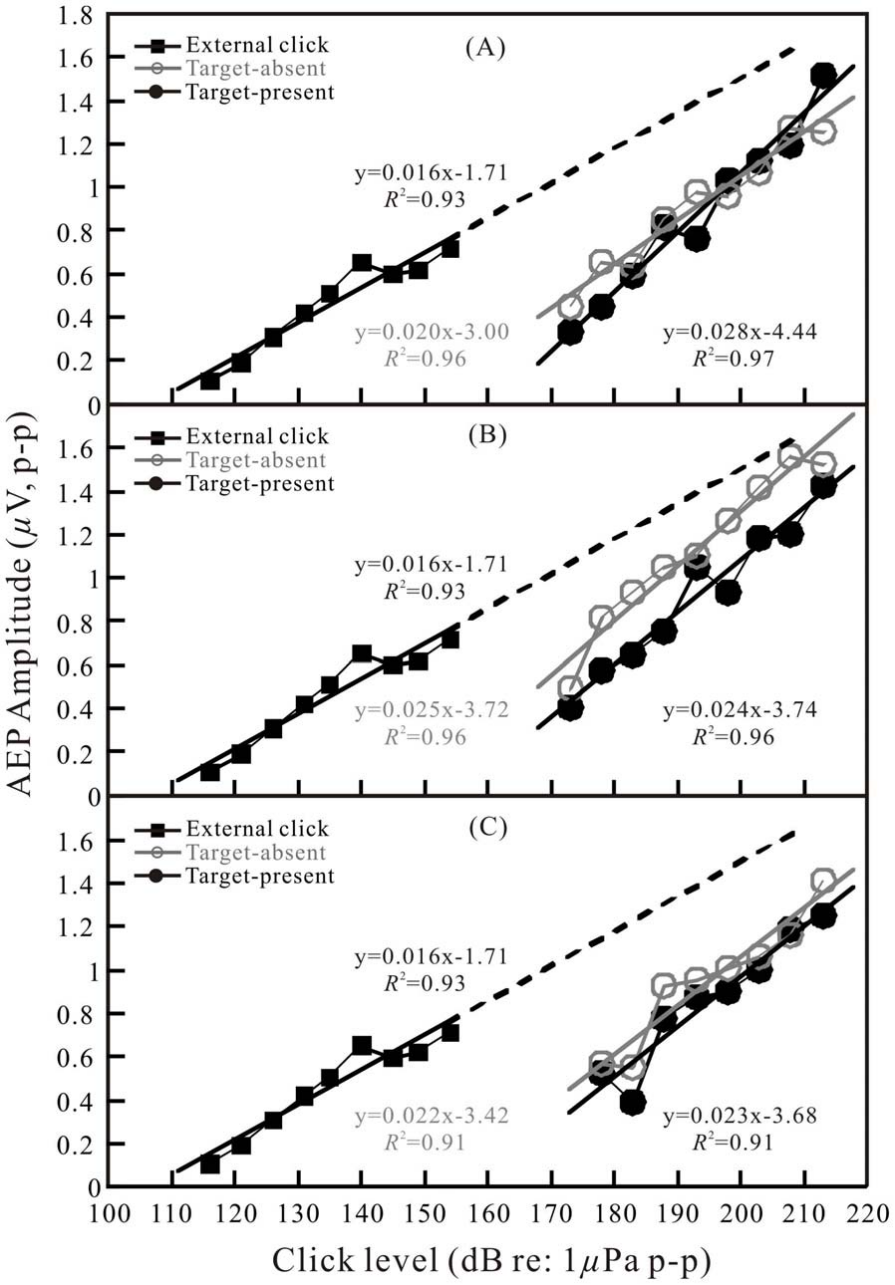
Same as in **Fig. 5** but for the target with −34-dB target strength.

AEPs evoked by external “dolphin-like” clicks with sound pressure levels (SPL, dB *re* 1 μPa peak-to-peak) between 116 and 155 dB (approximately 5-dB increments) directed toward the assumed “acoustic window” of the dolphin, 2.15 m from the sound projector, are presented in Fig. 4. AEPs evoked by external clicks with SPLs higher than 155 dB were not measured in order to avoid potential behavioral or physiological effects of loud external clicks. Fig. 4 shows that all of the records had a discernable AEP waveform located at approximately 3.5 to 6 ms after triggering. The AEP waveforms characterized by a couple of alternative positive-negative short waves were similar to the biosonar click-related AEP waveforms in Fig. 3. The AEP amplitudes increased with the sound pressure levels of the external clicks. The incline was most steep within the range of 116 to 140 dB (Figs. 5, 6, 7).

### AEP dependence on click level and comparison between external click-related and biosonar click-related AEP amplitudes

To quantitatively investigate the AEP dependence on click level and compare AEP amplitudes evoked by the emitted biosonar clicks with those evoked by the external clicks in the free field, amplitudes of the AEP waveforms with over 6 dB AEP-to-noise ratio were measured, corrected, and plotted as a function of click levels: SPLs for the external clicks and SLs for the emitted biosonar clicks (all as dB *re* 1 μPa peak-to-peak). The data are presented in Figs. 5, 6, 7 for different targets and target distances at both the target-present and target-absent conditions. Each function is shown along with a least-squares linear regression line, the corresponding equation and the correlation coefficient.

The dependence of the AEP amplitudes on click levels was significant (correlation test, *p*<0.05) for both external click-related AEPs and biosonar click-related AEPs (Figs. 5, 6, 7). The trends of AEP amplitude increase along with click levels in Figs. 5, 6, 7 were similar between external click-related AEPs and biosonar click-related AEPs. The external click-related AEP amplitude increased 0.016 μV for each 1-dB increment of the click sound pressure levels; the biosonar click-related AEP amplitude increased 0.014 to 0.029 μV for each 1-dB increment of the click source levels, depending on target type, distance and condition. However, the biosonar click-related AEP amplitude-versus-click SL functions were approximately shifted upward along the *click level* scale relative to the function of external click-related AEP amplitude-versus-click SPL.

In order to quantitatively characterize the approximate shifts of the amplitude-versus-level functions, we extrapolated the regression line for the external click-related AEP amplitude-versus-click SPL function up to around 1.6 μV for the AEP amplitude (Figs. 5, 6, 7). Subsequently, differences of click levels which evoked equal AEP amplitudes between the external click and the biosonar click were measured point-by-point along the regression lines at each level bin (±2.5 dB) of the biosonar clicks from 173±2.5 dB to 213±2.5 dB. The basic statistics of the measurement are presented in Table 2. The differences of click levels varied from 15.7 dB (−34 dB target at 3.5 m under the target-absent condition) to 35.9 dB (−34-dB target at 6.5 m under the target-present condition) on average.

**Table 2:** Basic statistics of the regression line shift (dB re: 1 μPa) measured by point-by-point click level differences along the regression lines between emitted biosonar click-related AEP amplitude-versus-click SL functions and external click-related AEP amplitude-versus-click SPL function, and comparison between target-present and target-absent conditions (Paired T-test, i.e., T-test for dependent samples).

Target	Target distance (m)	Target present condition	Regression line shift between biosonar click-related AEP amplitude-versus-click SL function and external click-related AEP amplitude-versus-click SPL function (dB)					Target present/absent difference: P-value (paired T-test)
			Min	Max	Mean	S.D.	Number of points	
−22 dB Target	2.0	present	10.1	41.6	**25.8**	10.8	9	P<0.0005^***^
		absent	13.9	42.7	**28.3**	9.8	9	
	3.5	present	29.8	33.3	**31.6**	1.2	9	p<0.0005^***^
		absent	15.7	28.2	**22.0**	4.3	9	
	6.5	present	34.0	35.0	**34.5**	0.3	9	p<0.0005^***^
		absent	23.1	28.1	**25.6**	1.7	9	
−28 dB Target	2.0	present	5.7	35.2	**20.4**	10.1	9	p = 0.2798
		absent	21.9	25.7	**23.8**	1.3	9	
	3.5	present	28.5	36.0	**32.3**	2.6	9	p<0.0005^***^
		absent	13.7	28.2	**21.0**	5.0	9	
	6.5	present	30.8	38.5	**34.6**	2.7	9	p<0.05^*^
		absent	26.7	31.7	**29.2**	1.7	9	
−34 dB Target	2.0	present	17.3	46.0	**31.7**	9.8	9	p = 0.4033
		absent	24.6	35.1	**29.8**	3.6	9	
	3.5	present	18.8	39.1	**29.0**	6.9	9	p<0.0005^***^
		absent	4.4	27.1	**15.7**	7.8	9	
	6.5	present	26.9	44.9	**35.9**	6.2	9	p<0.0005^***^
		absent	22.5	38.3	**30.4**	5.4	9	

*, and ***, difference is significant at p-level<0.05.

In some, but not all cases, there is also an obvious shift of AEP amplitude-versus-click SL functions between target-present and target-absent conditions (Fig. 5b and c, 6b, and 7b). The basic statistics of the measurement are presented in Table 2. In the majority of the cases, the shifts of the regression lines based on a point-by-point measurement between the target-present and target-absent conditions were significant (paired T-test, p<0.05).

Apart from extrapolation of the regression line, we also tried to obtain a direct estimate of the shift between the external click-related and biosonar click-related AEP amplitude-versus-level functions. It was possible within a range of biosonar click SLs of 178–183 dB and external click SPLs of 145–155 dB. In these ranges, both biosonar click-related AEPs and external click-related AEPs had similar amplitudes of approximately 0.6–0.7 μV. The shifts between the functions were estimated to be approximately 25 to 35 dB on average, and they varied with target type, distance, and condition (Figs. 5, 6, 7).

## Discussion

### Comparison of biosonar and external clicks

**Figure 8:**
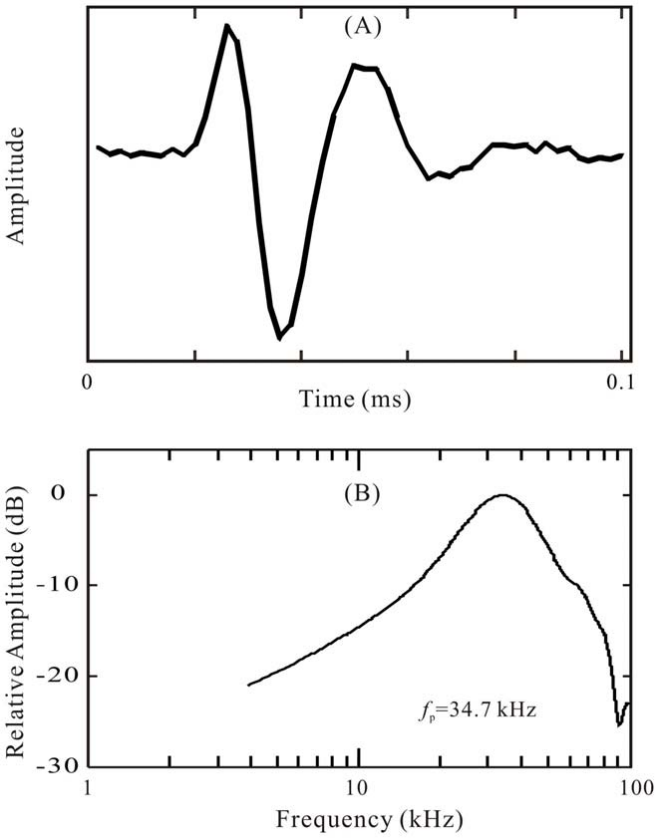
Waveform (a) and spectrum (b) of the external “dolphin-like” clicks.

A comparison of the external “dolphin-like” click (see Fig. 8) with the self-generated biosonar clicks (see Fig. 2) shows that both the waveform and spectrum were similar, except that the peak frequencies of the biosonar clicks of the levels from 170 to 180 dB were lower and those of 205 dB were higher than those of the external clicks.

### Hearing sensation levels of the emitted biosonar clicks

Since both the waveforms and spectra of the external clicks were similar to those of the biosonar clicks, and both the external clicks and biosonar clicks evoked AEPs with similar waveforms (Figs. 3 and 4), equal AEP amplitudes may be considered as an indication of equal hearing sensation levels of the two sound stimuli. Therefore, it was possible to evaluate hearing sensation levels of the biosonar clicks based on the AEP amplitudes evoked by the external clicks of known SPLs [18]. The relative shifts of the amplitude-versus-SL and amplitude-versus-SPL regression lines (see Figures 5, 6, 7) show that the hearing sensation level of a biosonar click with a certain SL is equal to that of an external click with SPL of 15.7 to 35.9 dB below the biosonar click SL, depending on target type, distance, and condition.

The external click-related AEPs were recorded at click SPLs up to 155 dB, at which the provoked AEP amplitude is around 0.7 μV. It was noted that there is a plateau in the external click-related AEP regression between the SPLs of 140 and 150 dB (Figs. 5, 6, 7). The reason is unclear in the present study. Nevertheless, based on the fact that the AEP amplitudes evoked by the biosonar clicks of similar waveform and spectrum increasing linearly up to approximately 1.6 μV, we assumed that the regression line of the external click-related AEP amplitude-versus-click level function could be extrapolated up to AEP amplitude of 1.6 μV. Besides, a direct comparison of biosonar click-related AEPs and external click-related AEPs was also done within a narrow range where amplitudes of the biosonar click-related AEPs and external click-related AEPs were overlapped (0.6–0.7 μV). Within the narrow range, the hearing sensation level of a biosonar click of a certain SL is equal to that of an external click with a SPL of approximately 25 to 35 dB below the sonar click SL. This direct comparison resulted in an evaluation somewhat different from, however overlapped by that obtained with the use of extrapolated regression lines. The difference between the two manners of evaluation may be a result of the very limited number of points available for direct comparison and slightly different slopes between the external click-related AEP amplitude-versus-click SPL function and biosonar click-related AEP amplitude-versus-click SL functions (Figs. 5, 6, 7). Nevertheless, the general result is the same between the two evaluation methods, i.e., the sensation level of a biosonar click is equal to that of an external click with SPL of approximately 16 to 36 dB on average below the biosonar click SL.

Rather similar ratios (−20 to −37 dB) between the hearing sensation level and SLs of biosonar clicks were found in a false killer whale [18]. These data may be assumed to indicate that both the bottlenose dolphin and false killer whale possess an effective protection system to isolate the self-produced biosonar beam from the animal's ears.

### Biosonar click sensation level dependence on echolocation conditions

The hearing sensation levels of the biosonar clicks varied depending on the type of target, distance, and condition (Figs. 5, 6, 7; Table 2). More specially, for all the examined target types at target distances of 3.5 and 6.5 m, the sensation levels were significantly different between the target-present and target-absent conditions (Table 2). At the same click SL, the sensation levels of the biosonar clicks during the target-absent condition was 5.4–13.3 dB higher than during the target-present condition. Although this difference was not statistically significant at a few combinations of target strength and distance (Table 2), the general trend of higher response at target-absent rather than at target-present conditions was obvious. This result would be expected if the dolphin were capable of adjusting her hearing response or sound-damping mechanisms according to the echolocation conditions as has been previously shown in a false killer whale [18]. In addition, the mean values of the biosonar click levels produced by the dolphin were consistently significantly higher at the target-absent conditions than those at the target-present conditions for all the examined target strengths and target distances (Table 1). Presumably, louder clicks required more energy. It may suggest that the dolphin also adjusted its biosonar production with more efforts to produce louder clicks during the target-absent condition. The behavioral and physiological significance of the adjustments of both hearing response and click production could be explained as that in the absence of a stronger echo, the increase of both hearing response and click intensity may be a dual-component way to increase the opportunity to acquire a weaker echo.

It was also noticed that in most cases the slope in the biosonar click-related AEP amplitude-versus-click SL function was steeper than that in the external click-related AEP amplitude-versus-click SPL function. This suggested that the louder biosonar clicks trend to have higher relative hearing sensation levels. Perhaps even though the biosonar clicks were generally similar to the external clicks, the waveforms and spectra of the biosonar clicks were slightly changing with click SLs, especially the peak frequencies shifting up with increasing amplitudes (Fig. 2), while the waveforms and spectra of the external clicks were constant. As it was known, the hearing sensitivity of the dolphin will change with frequencies of sound stimuli, the relative hearing sensation levels of the biosonar clicks with different click SLs and thus different peak frequencies may change. Perhaps, louder biosonar clicks with higher peak frequencies have higher relative hearing sensation levels, which resulted in the steeper slopes in the biosonar click-related AEP regression lines comparing with the external click-related AEP regression line. Alternatively, the dolphin might be actively improving her hearing sensation levels towards louder clicks.

## Materials and Methods

### Ethical Statement

This research was conducted under University of Hawaii Institutional Animal Care and Utilization Committee with approval protocol No. 93-005-17 to PEN and Marine Mammal Permit No. 978-1567 from the NMFS NOAA Office of Protected Resources to PEN. This is contribution number 1481 of the Hawaii Institute of Marine Biology.

### Subject

The experimental subject was an adult captive born female Atlantic bottlenose dolphin (*Tursiops truncatus*) named BJ, who was 24 years old at the time of the experiment. She was housed in a wire-net enclosure in the facilities of the Hawaii Institute of Marine Biology, Marine Mammal Research Program, Kaneohe Bay, Hawaii. The animal was trained to wear soft latex suction cups containing human EEG electrodes to pick up the evoked potentials. The animal performed echolocation tasks in which she swam into a hoop, ensonified and detected targets by echolocation, and reported the target presence or absence using a go/no-go reporting paradigm [19]. Three targets were used in this study. They were hollow aluminum cylinders with an outer diameter of 38 mm, an inner diameter of 25.4 mm, and lengths of 180, 90, and 46 mm. Their target strengths were −22, −28, and −34 dB, respectively, as measured by a short pulse produced by excitation of a 60-mm spherical piezoceramic transducer with 10-μs rectangular pulses. Previous investigations on peak frequencies of the biosonar produced by this animal indicated that the maxima of the distributions were between 40 and 50 kHz [20]. The AEP audiogram for this dolphin collected in 2001 and 2005 by using sinusoidally amplitude modulated (SAM) tone stimuli showed that her hearing thresholds abnormally increased at sound frequencies above 45 kHz; however, below this limit the thresholds were normal, and the animal was able to echolocate with performance levels over 95% [20].

### Experimental facilities and setup

#### AEP recording during echolocation

**Figure 9:**
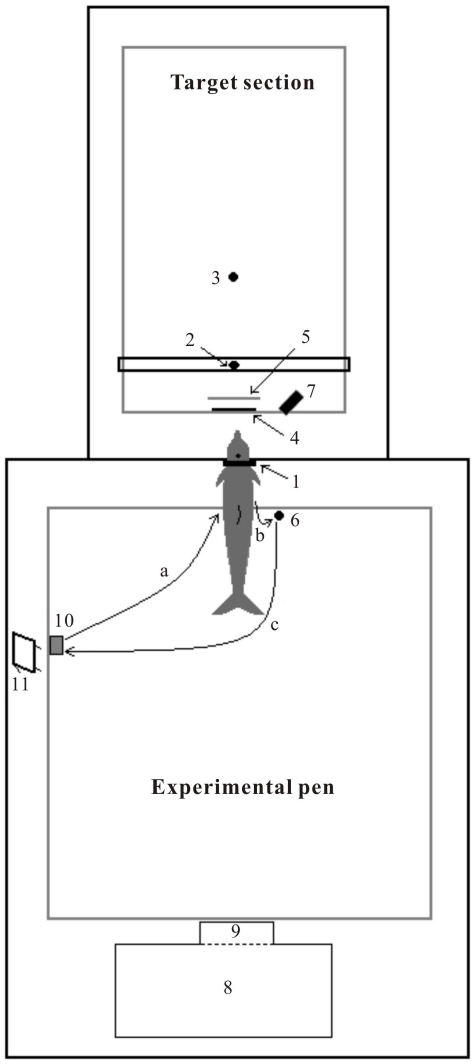
Experimental facilities and setup (top view). 1, Hoop station; 2, Recording hydrophone; 3, Target during echolocation sessions or transducer during AEP recording to external “dolphin-like” clicks; 4, Acoustic screen; 5, Visual screen; 6, Response ball; 7, Video camera; 8, Instrument shack; 9, Window; 10, Stationing pad; 11, Trainer position; a, the animal swam to the hoop station from the stationing pad; b, the animal got out of the hoop station to touch the response ball to report that the target was present; c, the animal swam back to the stationing pad.

**Figure 10:**
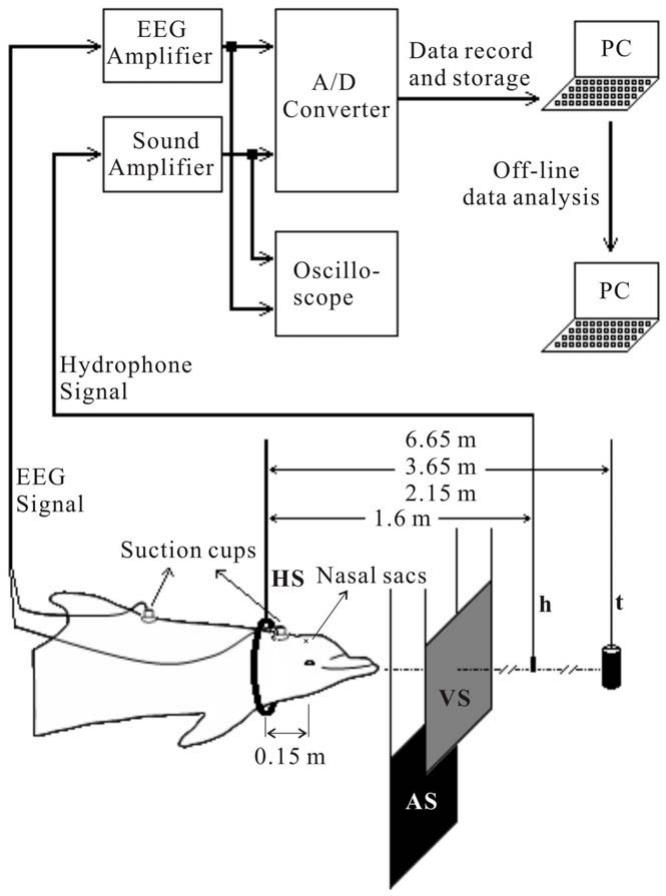
Schematic of the dolphin's relative position, data recording equipments, and data flow chart. AS, Acoustic screen; h, recording hydrophone; HS, Hoop station; PC, Laptop computer; t, Target during echolocation sessions or transducer during AEP recording to external “dolphin-like” clicks; VS, Visual screen.

The experimental facilities and setup were fully described in Li *et al.* [21] and described here as shown in Figs. 9 and 10. Briefly, the experimental enclosure consisted of two parts: (1) the experimental pen, and (2) the target presentation section. The experimental pen was an 8×10 m^2^ floating frame that bore a wire-net enclosure and was used to house the experimental subject during data collection. The target section was 6×8 m^2^ floating frame which served only to mount targets and did not bear wire net in order to avoid the production of confounding extra echoes. In the net divider separating the experimental pen and target section, there was a dolphin-sized circular opening bounded by a hoop (1 in Fig. 9). In front of the hoop, a RESON TC4013 hydrophone (Reson, Slangerup, Denmark; 2 in Fig. 9) was mounted on a beam at a distance of 1.6 m to the hoop (Fig. 10, approximately 1.45 m from the animal's nasal sacs) to record sounds emitted by the animal during her positioning in the hoop station. A target (3 in Fig. 9) was hung from a thin monofilament thread in the target section at distances of 2.15, 3.65 or 6.65 m to the hoop (Fig. 10, approximately 2, 3.5, or 6.5 m to the animal's nasal sacs). The target could be pulled up out of the water and lowered down into the water via pulleys from the instrument shack (8 in Fig. 9). Between the recording hydrophone and hoop station, there was a movable acoustic screen (4 in Fig. 9, AS in Fig. 10) and a fixed visual screen (5 in Fig. 9, VS in Fig. 10). During a trial, the acoustic screen was lowered which opened the space in front of the animal for echolocation. Near the hoop station, a response ball (6 in Fig. 9) was mounted above the water surface serving as a target-present response indicator. The animal's position in the station hoop was monitored through an underwater video camera (7 in Fig. 9) by both the experimenter in the instrument shack (8 in Fig. 9) and the trainer (position 11 in Fig. 9). When not in the hoop, the animal stationed at a foam stationing pad (10 in Fig. 9) waiting for the instructions from the trainer. The experimenter and trainer kept communication during experimental session through a window (9 in Fig. 9) of the instrument shack.

#### Recordings of AEP elicited by external “dolphin-like” clicks

The experimental facilities and setup were the same as in the echolocation sessions (Fig. 9), except that the target was replaced by an ITC-1032 spherical transducer (International Transducer Corporation, Santa Barbara, CA, USA) (3 in Fig. 9) at a distance of 2.15 m to the hoop for projecting the external clicks, and the hydrophone (2 in Fig. 9) and response ball (6 in Fig. 9) were removed.

### Experimental procedure, equipments and data collection

#### Data recording during echolocation

The experimental procedures were the same as those used by Li *et al.* [21] and briefly described here. Experimental sessions were typically conducted in the morning when fish was first offered. One session was conducted per day. Each session consisted of 50 trials, 25 target-present and 25 target-absent, randomly alternated using a modified Gellermann series [22].

Each session began with the trainer attaching suction-cup electrodes to the dolphin for AEP recording. Each trial was initiated with a hand signal to cue the animal to swim from the stationing pad to the hoop along trace ‘a’ in Fig. 9. The animal typically emitted 20 to 40 clicks during each trial. The biosonar clicks and the click-triggered AEPs were recorded by the click- and AEP-acquisition system (see below) operated by the experimenter in the instrument shack. The go response was required when the target was present and the no-go response was required when the target was absent. For the go response, the animal was required to back out of the hoop and follow trace ‘b’ in Fig. 9 to touch the response ball with her rostrum. The animal received a bridge whistle if her response was correct. The subject would then follow trace ‘c’ in Fig. 9 back to the stationing pad, receive the fish reward, and wait for the hand signal to begin the next trial. For the no-go response, instead of touching the response ball the animal was required to stay in the hoop for 6 s. If the response was correct, she received a bridge whistle and was required, following trace ‘c’ in Fig. 9, to return back to the stationing pad, receive her fish reward, and wait for the hand signal to begin the next trial. If the dolphin was incorrect in either a go or no-go trial, she was not given a fish reward and was required to return to the stationing pad waiting for the next trial.

The data recording equipment and flow chart are presented in Fig. 10. AEP responses were picked up by EEG electrodes which were gold-plated 10-mm disks (Grass Technologies, West Warwick, RI, USA) mounted within 60-mm silicon suction cups. The recording electrode was attached with conductive gel on the dorsal head surface, located midline, approximately 5–7 cm behind the blowhole. The reference electrode was also attached along with conductive gel on the animal's back near the dorsal fin. AEPs were led by shielded cables to an EEG amplifier (GRASS CP511 AC Amplifier, Grass Technologies) and amplified 20,000 times within a frequency band of 300 to 3,000 Hz. The amplified signal was monitored by an oscilloscope (Fluke 196C Scopemeter, Fluke Corporation, Everett, WA, USA) and input to a 16-bit analog-to-digital converter of a data acquisition card (NI USB-6251, National Instruments, Austin, TX, USA) connected to a standard laptop computer. Signals from the click-recording RESON TC4013 hydrophone were input to a signal amplifier (Krohn-Hite Model 3362 filter, Krohn-Hite Corporation, Brockton, MA, USA) and amplified by 20 dB within a frequency range of 1 to 200 kHz, monitored by the same oscilloscope, and led to another analog-to-digital converter of the same data acquisition card. Sampling rates were 25 kHz for the AEP-recording and 500 kHz for the click-recording.

The data collection process was controlled by the experimenter with a custom-made program designed with LabVIEW (National Instruments) running on the laptop computer. The program continuously monitored the click-recording input, and each time when the signal exceeded a predetermined triggering level (157 dB p-p *re*: 1 μPa), a 15-ms window of the EEG-recording channel and 0.1-ms window of the click-recording channel were recorded and stored in the memory of the laptop computer without averaging for off-line analysis. The click-recording window included a 0.02-ms pretrigger time.

#### AEP recording to external “dolphin-like” clicks

For this phase of the experiment, the animal was sent to the same hoop station by the trainer (position 11 in Fig. 9) after having the suction-cup electrodes attached for AEP recording. As soon as she took the proper position, external clicks were played through the transducer, and AEPs to these click stimuli were collected. After that, the animal was called back to the trainer for a fish reward.

The external clicks were produced with activation of the transducer by short rectangular pulses. The pulses were digitally generated by the same NI card and played through a 16-bit digital-to-analog converter, amplified by a power amplifier (Hewlett-Packard Agilent 465A, Palo Alto, CA, USA), and projected by the ITC-1032 spherical transducer. The resulting click waveform and spectrum are presented in Figure 8. Pilot measurements showed they were similar to typical biosonar clicks of the experimental subject in both waveform and spectrum [21].

The equipment for AEP collection included the same electrodes, amplifier, and data acquisition card as for data recording during echolocation. The amplifier gain, passband, and recording window for AEP recording were also same: 20,000 times, 300–3,000 Hz, and 15 ms, respectively. Unlike the AEP collection during echolocation, the card was programmed for on-line averaging to extract AEP from background noise. The averaging was triggered by the external clicks presented at a rate of 20/s. AEP was collected by averaging 1000 individual records.

The sound pressure levels (dB *re* 1 μPa peak-to-peak) of the external clicks were calibrated by using the same hydrophone (RESON TC4013) that was used for biosonar click recording during the echolocation sessions. The hydrophone was positioned at the center of the hoop station while the animal was not there, which is 2.15 m to the sound projector (transducer, Fig. 10). Thus, the position of the hydrophone was considered to be around the assumed “acoustic window” at the lower jaw of the subject [23].

### Data analysis

The AEP recordings triggered by the emitted biosonar clicks were sorted according to levels of the triggering click in 5-dB bins, from 160±2.5 dB to 215±2.5 dB *re* 1 μPa peak-to-peak. To extract low-amplitude AEPs from the background noise, an off-line averaging procedure was used [24]. In each bin, all the AEP records were averaged, thus each resulting AEP waveform corresponded to a certain biosonar-click level with a tolerance of ±2.5 dB [18]. Only the bins with AEP-to-noise ratio over 2 (i.e., 6 dB) were included for final analysis; these were bins from 170±2.5 dB to 210±2.5 dB *re* 1 μPa peak-to-peak. The starting part of the obtained averaged AEP records appeared to be contaminated by some artifacts; therefore, the first 15 sampling points of each AEP record (0.6 ms on the time scale) were ignored. A high-pass zero-phase shift digital filtering with a cutoff frequency of 800 Hz was used for the rest of the record (Li *et al.*, 2011). This process slightly changed the AEP waveform and reduced the low-frequency AEP components but was considered as acceptable for comparison of responses analyzed in the same way [21], [24]. The peak-to-peak amplitudes of the averaged and filtered AEPs were then measured. Even after the filtering, the potential addition of background noise to the response might increase the measured amplitude within the AEP window, thus resulting in overestimate of AEP amplitude. To mitigate the effect of the potential addition of background noise, the measured peak-to-peak AEP amplitude was corrected by subtracting the noise power from the response power using the formula:
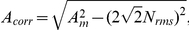
where *A_m_* is the measured peak-to-peak amplitude in the AEP response window, *N_rms_* is noise RMS within a response-free window in the same averaged and filtered record, *A_corr_* is the corrected peak-to-peak amplitude, and the factor of 2√2 relates peak-to-peak to RMS values [25].

The on-line averaged AEPs to external clicks were filtered in the same way as AEPs during echolocation. The corresponding peak-to-peak AEP amplitudes were measured and then corrected for background noise in the same manner as biosonar click-related AEPs.
